# Supplementary material for: Relationship between Physicochemical Characteristics and Pathogenic Leptospira in Urban Slum Waters
Source: Trop Med Infect Dis. 2020 Sep 16;5(3):146. doi: 10.3390/tropicalmed5030146 (PMC7558472; doi:10.3390/tropicalmed5030146)
Supplement: Supplementary file 1 [file tropicalmed-05-00146-s001.pdf]

SUPLEMENTARY TABLES

Table S1. Distribution of collected samples, presented by collection period and water type.

	Sewage Water		Standing Water		
Period	Collected samples	Positive samples	Collected samples	Positive samples	Positive samples/ samples collected
July	84	38 (45%)	70	35 (50%)	73/154 (47%)
January	82	12 (15%)	48	22 (46%)	34/130 (26%)
Total	166	50 (30%)	118	57 (48%)	107/284 (38%)

Table S2. Physicochemical parameters (mean and standard deviation) and concentration of pathogenic Leptospira (geometric mean and count range among positive samples) measured in the standing and sewage water samples collected in Pau da Lima.

	**Overall**	**Sewage**	**Standing**	***p***
Temperature (°C)	25.7 ± 2.21	25.9 ± 2.05	25.3 ± 2.38	0.01
pH	7.2 ± 0.45	7.3 ± 0.32	7.1 ± 0.56	<0.01
Turbidity (NTU)	298 ± 223	229 ± 151	395 ± 270	<0.01
Total dissolved solids (TDS) (mg/L)	530 ± 272	652 ± 219	359 ± 247	<0.01
Electrical Conductivity (μS)	0.98 ± 0.47	1.20 ± 0.35	0.67 ± 0.44	<0.01
Salinity (‰)	0.26 ± 0.25	0.37 ± 0.20	0.12 ± 0.23	<0.01
Leptospira concentration (GEq/mL)	194 ± 205.7	181 ± 153.5	205 ± 243.3	0.94

Table S3. Estimated regression parameters in the bivariate logistic (Odds ratio and confidence interval) and linear (coefficient and confidence interval) models on the probability of finding a positive sample and log10 concentration of Leptospira, respectively. Analysis of the interaction of chemical physical parameters by water type. (***) $p < 0.01$, (**) $p < 0.05$, (*) $p < 0.1$.

	**Logistic Model**			**Linear Model**		
	Odds Ratio	*95% CI*	*p*	*Coeficiente*	*CI*	*p*
Temperature	0.86	0.76–0.96	**0.009**	−0.04	−0.07–−0.00	**0.05**
Temperature :Water	1.02	1.01–1.04	**0.011**	0	−0.01–0.00	0.692
pH	0.45	0.26–0.79	**0.006**	0.23	0.06–0.40	**0.01**
pH: Water	1.09	1.02–1.17	**0.01**	0.01	−0.01–0.03	0.491
Turbidity	1	1.00–1.00	0.132	0	−0.00–0.00	0.125
Turbidity: Water	1	1.00–1.00	**0.019**	0	−0.00–0.00	0.221
TDS	1	1.00–1.00	**<0.001**	0	−0.00–0.00	0.822
TDS: Water	1	1.00–1.00	**0.044**	0	−0.00–0.00	0.82
Salinity	0.27	0.09–0.74	**0.013**	0.28	−0.06–0.62	0.101
Salinity: Water	0.63	0.29–1.29	0.221	0.24	0.02–0.45	**0.03**
EletrConduc	0.48	0.28–0.82	**0.009**	0.19	0.01–0.37	**0.04**
EletrConduc: Water	0.99	0.66–1.44	0.951	0.16	0.05–0.27	**0.01**
